# Altered Endometrial Memory T-Cell Profiles During the Window of Implantation in Women with Previous Miscarriage

**DOI:** 10.3390/biomedicines13112800

**Published:** 2025-11-17

**Authors:** Dimitar Parvanov, Rumiana Ganeva, Margarita Ruseva, Maria Handzhiyska, Jinahn Safir, Lachezar Jelezarsky, Dimitar Metodiev, Georgi Stamenov, Savina Hadjidekova

**Affiliations:** 1 Department of Research, Nadezhda Women’s Health Hospital, 1330 Sofia, Bulgaria; 2 Department of Clinical Pathology, Nadezhda Women’s Health Hospital, 1330 Sofia, Bulgaria; 3 Department of Obstetrics and Gynecology, Nadezhda Women’s Health Hospital, 1330 Sofia, Bulgaria; 4 Department of Medical Genetics, Medical University of Sofia, 1431 Sofia, Bulgaria

**Keywords:** human endometrium, window of implantation, memory T cells, central memory T cells (TCM), effector memory T cells (TEM), tissue-resident memory T cells (TRM), terminally differentiated effector memory T cells (TEMRA), miscarriage, reproductive immunology

## Abstract

**Aim:** This study aimed to characterize and compare the composition of central (TCM), effector (TEM), tissue-resident (TRM), and terminally differentiated (TEMRA) memory T cells in mid-luteal endometrium during the window of implantation (WOI) in women with and without a previous miscarriage. **Methods:** Stromal lymphocytes from endometrial samples (P + 5) were analyzed by multicolor flow cytometry to quantify total, CD4^+^ and CD8^+^ TCM (CD45RA^−^CCR7^+^), TEM (CD45RA^−^CCR7^−^), TRM (CD69^+^), and TEMRA (CD45RA^+^CCR7^−^) subsets. Participants were grouped as having no previous miscarriage (*n* = 38) or ≥1 previous miscarriage (*n* = 33), and the relative distribution of these memory subsets was compared between groups. Correlations, PCA and logistic regression were used to assess global memory network organization. **Results:** Women with prior miscarriage exhibited higher TCM proportions among total and CD8^+^ lymphocytes (*p* < 0.01), alongside lower CD8^+^ TEM (*p* = 0.02) and higher CD4^+^ TEM (*p* = 0.01). TRM showed a mild, non-significant increase (*p* = 0.18), while TEMRA remained stable. TRM correlated positively with both TCM (r = 0.51) and CD4^+^ TEM (r = 0.40), indicating coordinated organization among memory subsets. Multivariate analyses (PCA and logistic regression) confirmed these trends and identified the TCM/TEM ratio as the most discriminative parameter. **Conclusions:** Endometrial memory T-cell composition during the WOI differs in women with miscarriage history, characterized by central memory expansion and reduced effector memory proportions, with parallel increases in tissue-resident cells. These changes suggest persistent remodeling of the local immune memory network toward a long-lived, less differentiated phenotype that may influence implantation readiness in subsequent cycles.

## 1. Introduction

Successful embryo implantation depends on a receptive endometrial microenvironment established during the window of implantation (WOI). This specific period, typically five to seven days after ovulation or progesterone exposure, is characterized by coordinated crosstalk between stromal, epithelial, and immune cells that ensures tolerance to the semi-allogeneic embryo while maintaining protection against pathogens [1,2,3]. Dysregulation of this immune dialogue has been implicated in implantation failure, recurrent pregnancy loss, and infertility. Among immune components, T cells are fundamental in mediating local immune homeostasis and facilitating maternal–fetal tolerance. Traditionally, regulatory T cells (Tregs) are recognized as central mediators of immune tolerance at implantation, as they suppress excessive effector immunity and support trophoblast invasion and vascular adaptation [4,5]. However, less is known about how other T-cell subsets, particularly memory phenotypes, contribute to endometrial immune readiness.

Within the T-cell compartment, memory phenotypes contribute immunological surveillance and may shape the local readiness of the immune system. In reproductive immunology, the concept of immune memory is gaining traction: Kieffer et al. (2019) proposed that a delicate balance of memory T-cell subsets appears to be required for reproductive success, by facilitating tolerance without losing capacity for effective immune response [6]. These memory T-cell subsets are generally classified according to the coordinated expression of CD45RA, CCR7, CD69, and CD103, distinguishing central memory (TCM; CD45RA^−^CCR7^+^), effector memory (TEM; CD45RA^−^CCR7^−^), tissue-resident memory (TRM; CD69^+^), and terminally differentiated effector memory cells re-expressing CD45RA (TEMRA; CD45RA^+^CCR7^−^) phenotypes [7,8,9,10].

Tissue-resident memory T cells (TRM) are emerging as key players in the endometrium due to their ability to persist locally and provide rapid in situ immune responses without recirculation. TRM phenotypes have been identified at the maternal–fetal interface and implicated in pregnancy immunoregulation [11]. In the human endometrium, Southcombe et al. (2017) reported altered CD8 and CD69 expression in women with recurrent miscarriage, suggesting disruption of the resident memory compartment [12]. However, no study has systematically compared all four major memory T-cell subsets—TCM, TEM, TRM, and TEMRA—in the endometrium during the implantation window.

The long-term immunological impact of a prior miscarriage on endometrial memory T-cell composition is poorly understood. Pregnancy loss may leave persistent immunological imprints, potentially affecting subsequent implantation cycles. Therefore, the aim of this study was to characterize the relative composition of central (TCM), effector (TEM), tissue-resident (TRM), and terminally differentiated (TEMRA) memory T-cell subsets in the human endometrium during the window of implantation, and to compare their proportional abundance between women with and without a history of miscarriage.

## 2. Materials and Methods

### 2.1. Study Design and Participants

This observational comparative study included 71 women undergoing fertility evaluation at Nadezhda Women’s Health Hospital (Sofia, Bulgaria) between October 2024 and July 2025. Participants were stratified into two groups: control group (no history of miscarriage, *n* = 38) and miscarriage group (≥1 previous miscarriage, *n* = 33). All women were in good general health, had regular menstrual cycles, and had not received hormonal or immunomodulatory treatment for at least three months prior to sampling. The study was conducted in accordance with the Declaration of Helsinki and approved by the institutional ethics committee (protocol No. 45/07 approved on 7 December 2020). Written informed consent was obtained from all participants.

### 2.2. Endometrial Biopsy Collection

Endometrial biopsies were collected during the mid-luteal phase, five days after progesterone initiation (P + 5), corresponding to the window of implantation (WOI). In both groups, biopsies were timed to P + 5 after exogenous progesterone administration to synchronize endometrial development and ensure sampling during the implantation window. Sampling was performed with a Pipelle^®^ catheter (CooperSurgical, Trumbull, CT, USA), and tissue was immediately placed in ice-cold phosphate-buffered saline (PBS) for lymphocyte isolation. The timing of the luteal phase was verified by serum progesterone (P4) and estradiol (E2) measurements and by histological confirmation of secretory morphology and receptor (PR/ER) immunostaining in representative sections.

### 2.3. Isolation of Stromal Lymphocytes

Fresh endometrial tissue was processed immediately after collection. Stromal lymphocytes were isolated by gentle mechanical dissociation through repeated passage of the tissue through a sterile syringe and needle in cold PBS. The resulting suspension was filtered through a 35 µm nylon mesh (Falcon, Teterboro, NJ, USA) and centrifuged at 400× *g* for 10 min. The cell pellet was resuspended in PBS with 2% fetal bovine serum (FBS; Gibco, Thermo Fisher Scientific, Waltham, MA, USA) and used for flow cytometric analysis within 2 h. This mechanical approach was selected to minimize epitope loss and preserve cell viability.

### 2.4. Flow Cytometry Panel and Gating Strategy

Endometrial lymphocytes were stained with fluorochrome-conjugated monoclonal antibodies: CD45-PerCP (clone 2D1, ImmunoTools (Friesoythe, Germany), #21810455), CD3-PE (clone UCHT1, ImmunoTools, #21620034X2), CD4-BV425 (clone RPA-T4, BioLegend (San Diego, CA, USA), #300532), CD8-APC-Cy7 (clone RPA-T8, BioLegend, #344714), CD45RA-FITC (clone MEM-56, ImmunoTools, #21279453), CCR7-AF647 (clone 2-L1-A, Elabscience (Houston, TX, USA), #E-AB-F1159M), CD103-APC (clone Ber-ACT8, Biolegend #350216) and CD69-FITC (clone FN50, ImmunoTools, #21620693).

Stained samples were acquired on a BD FACSLyric™ flow cytometer (BD Biosciences, San Jose, CA, USA) operated with FACSuite™ software, v.1.5 (BD Biosciences). At least 100,000 lymphocyte events were collected per sample. Lymphocytes were gated based on forward/side scatter properties and CD45 expression. Antibody titration and compensation were optimized using single-stain controls. Viability was >95% as assessed by FSC/SSC scatter; no viability dye was applied due to immediate analysis of fresh tissue. Instrument settings were standardized across runs using BD CS&T beads.

**Figure A1:**
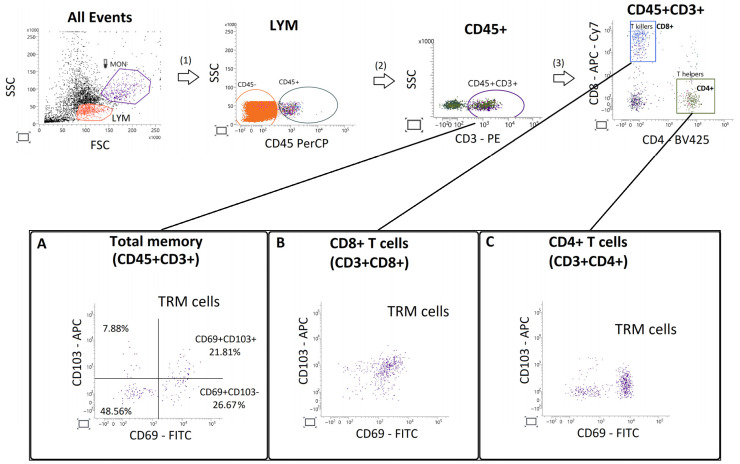
Gating strategy and validation of CD103 expression in endometrial T cells. Representative flow cytometry gating sequence (**top row**) showing selection of lymphocytes (LYM) based on forward and side scatter (FSC/SSC), followed by gating of CD45^+^ leukocytes, CD45^+^CD3^+^ T cells, and subsequent separation into CD8^+^ and CD4^+^ subsets. Panels (**A**–**C**) depict bivariate plots of CD69 (FITC) versus CD103 (APC) expression in total CD45^+^CD3^+^ T cells (**A**), CD8^+^ T cells (**B**), and CD4^+^ T cells (**C**). Horizontal and vertical lines mark fluorescence thresholds determined by fluorescence-minus-one (FMO) controls. The proportion of CD69^+^CD103^+^ cells was low and inconsistent across compartments (example shown in **A**), with extensive overlap of CD103 intensity between CD69^+^ and CD69^−^ populations. These observations confirm that CD103 did not provide additional discriminatory power for TRM identification in endometrial tissue.

Memory T-cell subsets were defined as follows: central memory (TCM; CD45RA^−^CCR7^+^)), effector memory (TEM; CD45RA^−^CCR7^−^), tissue-resident memory (TRM; CD69^+^), and terminally differentiated effector memory (TEMRA; CD45RA^+^CCR7^−^). TRM were classified by CD69 expression alone because CD103 staining in pilot samples (*n* = 10) was highly variable and failed to delineate a stable CD103^+^ compartment in endometrial lymphocytes; therefore CD103 was omitted from the final gating (Figure A1).

**Figure A2:**
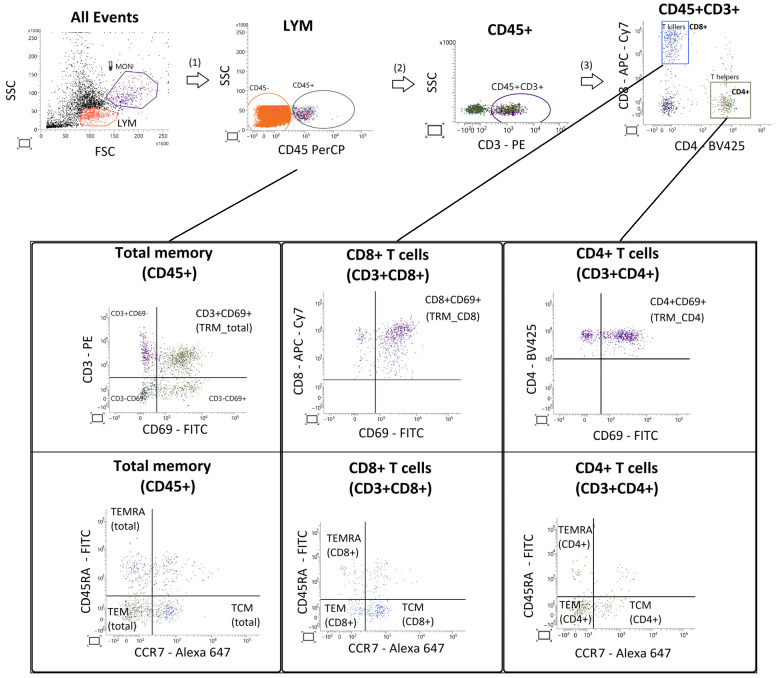
Gating strategy for identification of endometrial memory T-cell subsets. Lymphocytes were selected based on FSC/SSC characteristics and CD45 expression. Within the CD45^+^CD3^+^ population, helper (CD4^+^) and cytotoxic (CD8^+^) T cells were identified. Tissue-resident memory T cells (TRM) were defined as CD69^+^ cells, while central memory (TCM), effector memory (TEM), and terminally differentiated effector memory (TEMRA) subsets were defined according to CD45RA/CCR7 expression.

Each subset was evaluated either as a percentage of all CD45^+^ lymphocytes (Total TCM, TEM, TRM and TEMRA), or within CD3^+^CD4^+^ and CD3^+^CD8^+^ T-cell compartments. Specifically, CD4-derived memory subsets (TCM_CD4, TEM_CD4, TRM_CD4, TEMRA_CD4) represent percentages of total CD3^+^CD4^+^ T helper cells, while CD8-derived subsets (TCM_CD8, TEM_CD8, TRM_CD8, TEMRA_CD8) are expressed as percentages of total CD3^+^CD8^+^ cytotoxic T cells. The sequential gating hierarchy (CD45^+^ lymphocytes → CD3^+^ T cells → CD4^+^/CD8^+^ compartments → memory subsets) is illustrated in Figure A2.

### 2.5. Statistical Analysis

Statistical analyses were performed using SPSS v21 (IBM Corp., Armonk, NY, USA) and R v4.4.2 (R Foundation for Statistical Computing, Vienna, Austria). Data normality was assessed by the Shapiro–Wilk test. Group comparisons were conducted using the Student’s *t*-test or the Mann–Whitney U test, as appropriate. Correlations between T-cell subsets were evaluated with Spearman’s rank coefficient. Results are presented as mean ± SD or median [IQR], and *p* < 0.05 was considered statistically significant.

The compositional structure of CD45RA/CCR7-defined subsets (TCM, TEM, TEMRA) was analyzed within separate closures (total CD3^+^, CD4^+^, CD8^+^) using centered log-ratio (CLR) transformation (compositions R package, v. 4.4.2). TRM (CD69^+^) frequencies were analyzed independently, outside the CLR framework and correlated with CLR-based log-ratios. Logistic regression models were applied to test independent associations between memory subset ratios and miscarriage status. Variables were standardized (z-scores), and odds ratios (OR) with 95% confidence intervals (CI) were obtained by exponentiating regression coefficients. Discrimination was assessed by ROC curves and AUC (pROC package, R), and multivariate separation by PCA with PERMANOVA.

## 3. Results

### 3.1. Participant Characteristics

A total of 71 women were included in the study, comprising 38 controls with no previous miscarriage and 33 women with at least one miscarriage. The groups did not differ significantly in age, body mass index (BMI), or serum hormone levels (P4 and E2) measured at the time of biopsy (Table 1). All biopsies displayed histological features consistent with the mid-luteal phase and implantation window, confirmed by endometrial morphology and immunohistochemical assessment of estrogen and progesterone receptor expression.

### 3.2. Memory T-Cell Subsets in the Endometrium

**Figure 1:**
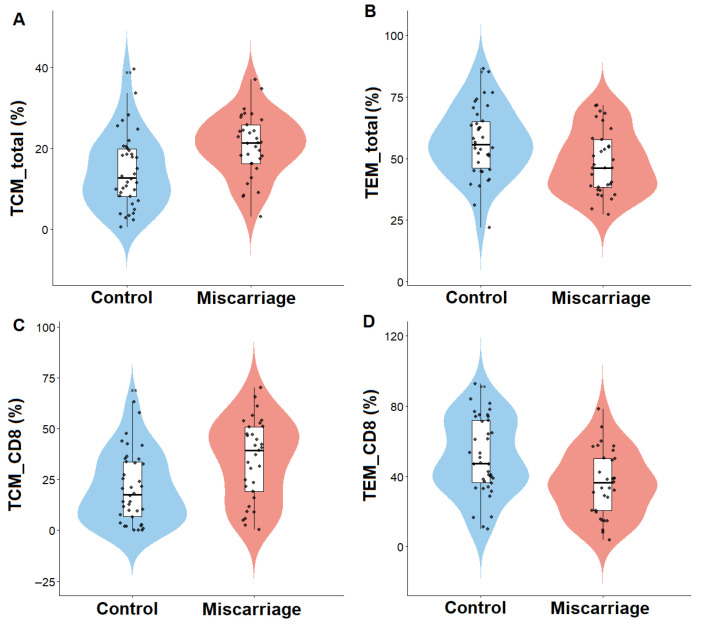
Distribution of endometrial memory T-cell subsets in women with (miscarriage group) and without (control group) previous miscarriage. Violin plots show the relative abundance of (**A**) total central memory T cells (TCM_total), (**B**) total effector memory T cells (TEM_total), (**C**) CD8^+^ central memory T cells (TCM_CD8), and (**D**) CD8^+^ effector memory T cells (TEM_CD8). Lines represent median and interquartile range. *p* < 0.05 was considered significant.

The relative abundance of endometrial memory T-cell subsets in the two study groups is summarized in Table 2 and illustrated in Figure 1.

Women with previous miscarriage showed a significantly higher proportion of central memory T cells (TCM) within total CD45^+^ lymphocytes (*p* = 0.003), and within the CD8^+^ compartment (*p* = 0.002). In contrast, effector memory T cells (TEM) were reduced in the miscarriage group, affecting both total TEM (*p* = 0.014) and CD8^+^ TEM cells (*p* = 0.003). Tissue-resident memory T cells (TRM) also showed a trend toward higher frequencies in the miscarriage group (47.2 ± 16.7 vs. 41.7 ± 15.2, NS), suggesting partial enrichment of the resident memory compartment despite the lack of statistical significance. Within the CD4^+^ compartment, TEM cells were elevated in women with miscarriage (*p* = 0.011), while no significant differences were observed for CD4^+^ TCM, TRM, or TEMRA subsets.

Overall, TRM and TEMRA populations remained comparable between groups across all analyzed compartments.

### 3.3. Correlations Among Memory T-Cell Subsets

**Figure 2:**
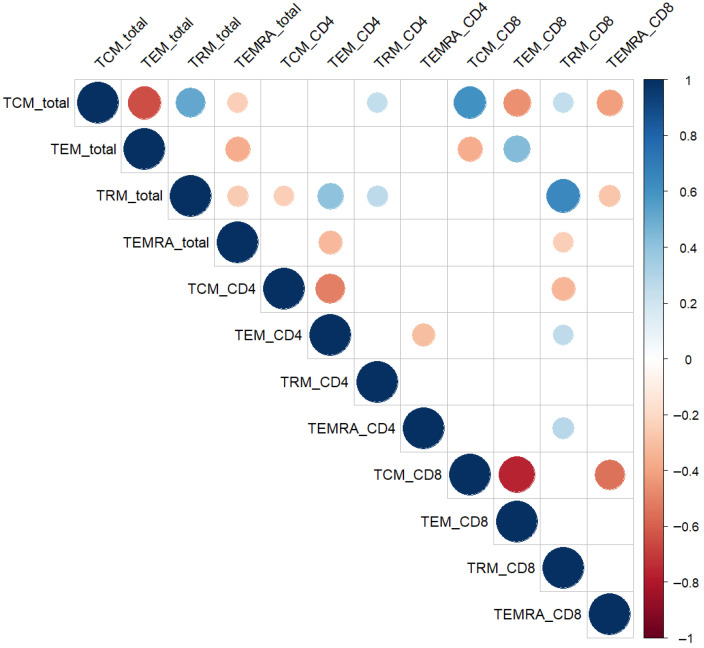
Spearman correlation matrix of endometrial memory T-cell subsets. Circle size and color represent the strength and direction of the correlation (red = positive, blue = negative). Only statistically significant correlations (*p* < 0.05, Spearman’s test) are displayed. Abbreviations: TCM—central memory; TEM—effector memory; TRM—tissue-resident memory; TEMRA—terminally differentiated effector memory T cells.

Spearman correlation analysis demonstrated coordinated relationships among distinct memory T-cell compartments in the endometrium (Figure 2).

A moderate positive correlation was observed between total TRM% and both total TCM% (r = 0.51, *p* < 0.01) and TEM_CD4% (r = 0.40, *p* < 0.01), suggesting coregulation and functional alignment between tissue-resident and circulating memory phenotypes.

Conversely, TEMRA subsets showed divergent association patterns with TRM populations. TEMRA_CD8 correlated negatively with TRM_CD8 (r = −0.55, *p* < 0.01), whereas TEMRA_CD4 displayed a weak positive correlation with TRM_CD8 (r = 0.27, *p* = 0.02). In addition, total TEMRA% was inversely associated with total TRM% (r = −0.26, *p* = 0.03).

These relationships provided the rationale for subsequent multivariate analyses aimed at capturing coordinated changes across memory T-cell compartments.

### 3.4. Multivariate Pattern Analysis of Endometrial Memory T Cells

#### 3.4.1. Global Differences Across Total Memory Subsets

**Figure 3:**
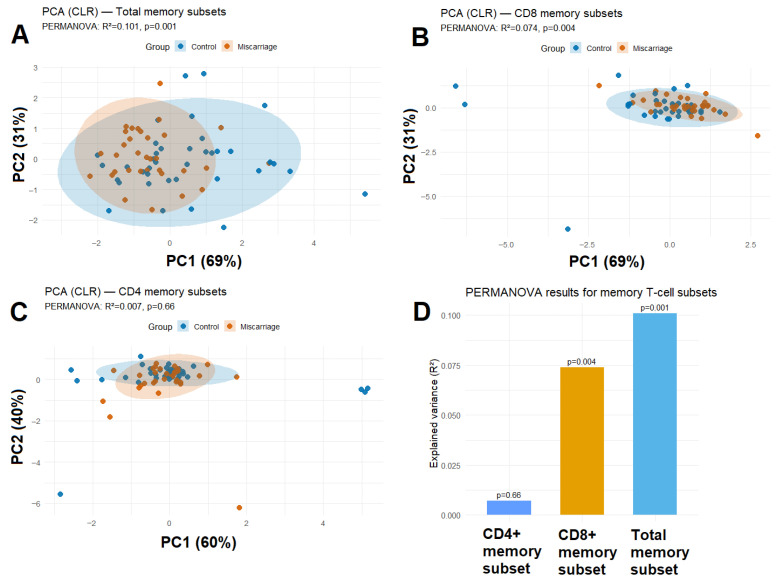
PCA (CLR) of (**A**) total memory subsets, (**B**) CD8^+^ memory subsets and (**C**) CD4^+^ memory subsets showing partial separation between groups. Shaded ellipses represent 95% confidence regions. (**D**) PERMANOVA results showing effect sizes (R^2^) and *p*-values for total, CD4^+^, and CD8^+^ memory subsets.

Principal component analysis (PCA) of CLR-transformed frequencies revealed a modest but statistically significant separation between women with and without previous miscarriage (PERMANOVA R^2^ = 0.101, *p* = 0.001; Figure 3A). The first two principal components explained 69% and 31% of total variance, respectively.

#### 3.4.2. Subset-Specific PCA for CD4^+^ and CD8^+^ Memory Profiles

When the analysis was restricted to CD8^+^ T-cell subsets, a trend toward group separation was observed (PERMANOVA R^2^ = 0.074, *p* = 0.004; Figure 3B). This finding aligns with the univariate and regression results, highlighting cytotoxic T-cell memory polarization as the most sensitive parameter. In contrast, CD4^+^ T-cell subsets showed substantial overlap between groups (PERMANOVA R^2^ = 0.007, *p* = 0.66; Figure 3C), indicating that helper memory differentiation remains largely unaffected. Thus, CD8^+^ memory differentiation appears more dynamically reshaped after miscarriage than CD4^+^ helper subsets.

#### 3.4.3. Integration with Regression Modeling

**Figure 4:**
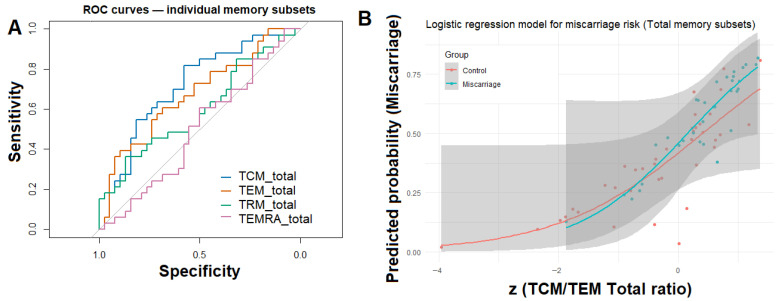
Logistic modeling and ROC curve analysis for total endometrial memory T-cell subsets. (**A**) Receiver operating characteristic (ROC) curves for individual total memory subsets (TCM_total, TEM_total, TRM_total, and TEMRA_total) illustrating their relative ability to discriminate between women with and without previous miscarriage. (**B**) Logistic regression model demonstrating the predicted probability of miscarriage as a function of standardized TCM/TEM_total ratio.

To further evaluate the discriminative potential of memory T-cell composition, individual ROC curves were constructed for the four major memory subsets (TCM, TEM, TRM, and TEMRA) expressed as percentages of total CD45^+^ lymphocytes (Figure 4A). Among these, TCM_total displayed the highest area under the curve (AUC = 0.72), followed by TEM_total (AUC = 0.67), whereas TRM_total and TEMRA_total showed lower predictive performance (AUC < 0.60).

To integrate these findings, a multivariable logistic regression model was constructed including the TCM/TEM_total ratio, TRM_total, age, and BMI as covariates. Only the TCM/TEM_total ratio remained significantly associated with miscarriage status (β = 1.01, *p* = 0.01; OR = 2.72, 95% CI: 1.40–6.22), indicating that compositional memory balance, rather than absolute subset frequencies, better distinguishes women with prior miscarriage. The model achieved an AUC = 0.75, indicating moderate discriminatory ability (Figure 4B).

A similar trend was observed within the CD8^+^ T-cell compartment (AUC = 0.76), reinforcing the robustness of this memory polarization signature. A parallel analysis restricted to the CD8^+^ memory compartment yielded a similar pattern, where the TCM/TEM_CD8 ratio emerged as the strongest independent predictor (β = 1.51, *p* = 0.02; OR = 4.53, 95% CI: 1.60–16.53), while other covariates were not significant.

## 4. Discussion

This study provides new insights into the organization of endometrial memory T-cell compartments during the window of implantation (WOI) in women with a history of miscarriage. We observed a selective expansion of central memory T cells (TCM), accompanied by reciprocal redistribution of effector (TEM) and tissue-resident (TRM) populations, whereas the terminally differentiated TEMRA subset remained stable. These findings suggest that the endometrial memory network maintains a coordinated, hierarchical structure rather than stochastic variation, in which changes in one compartment are mirrored by compensatory adjustments in others. Such compositional remodeling may represent immune adaptation following pregnancy loss, resulting in persistent recalibration of local immune memory and readiness for implantation.

The increase in TCM proportions and reduction in TEM frequencies observed in the miscarriage group indicate a bias toward a “central memory–dominant” phenotype in the endometrium. TCM cells possess self-renewal capacity, longevity, and proliferative potential upon antigenic restimulation, whereas TEM cells mediate rapid effector responses and migrate to inflamed tissues [13,14]. This polarization toward TCM dominance may represent a long-term shift toward immune surveillance rather than immediate effector activation, suggesting a memory profile oriented toward recall rather than acute inflammation.

Similar alterations in T-cell memory composition have been described in peripheral blood and mucosal tissues of women with reproductive failure [6,15,16]. In agreement with our results, Kang et al. reported enrichment of CD8^+^ central memory cells in the endometrium of women with recurrent miscarriage, indicating that miscarriage history may reshape the cytotoxic memory hierarchy. Parallel observations in other mucosal and barrier tissues, including lung, intestine, and cervix, show that repeated antigenic exposures promote enrichment of central-type memory cells [17,18,19], suggesting that a similar imprinting mechanism may operate in the uterine microenvironment. An enrichment of central memory T cells within the endometrium may also have functional consequences during the implantation window. In mucosal tissues, dominance of central-type memory phenotypes has been linked to enhanced immune surveillance and controlled cytokine production, favoring tissue homeostasis over acute inflammation [17,18,19]. Translating these parallels to the endometrium, a similar shift could reshape the local cytokine and chemokine milieu, influencing trophoblast migration, vascular remodeling, and stromal-epithelial communication during implantation. Such a balance might ensure immune readiness while preventing excessive effector activation that could compromise embryo invasion.

Principal component and logistic regression analyses further supported this interpretation. The multivariate PCA based on CLR-transformed memory subset frequencies revealed modest but statistically significant separation between groups, indicating that miscarriage is accompanied by coordinated remodeling of the T-cell memory network rather than isolated shifts in single subsets. In line with this, logistic regression identified the TCM/TEM ratio as the strongest independent discriminator between groups, with an odds ratio of approximately 2.7. This finding highlights that the balance between central and effector memory cells, rather than their absolute frequencies, captures the immunologic imprint of prior pregnancy loss. Comparable associations have been described in chronic viral infections and autoimmune disorders, where a skewed TCM/TEM ratio reflects altered immune homeostasis and reduced effector competence [20,21].

Although cytokine profiling was not performed in this cohort, previous studies have demonstrated that IL-15 and IL-7 are critical for memory T-cell maintenance and TGF-β in tissue residency. For example, IL-7 and IL-15 together maintain CD8^+^ TRM and IL-7 alone supports CD4^+^ TRM persistence [22]. Additionally, T-box transcription factors have been shown to cooperate with IL-15 and TGF-β to regulate CD8^+^CD103^+^ TRM development and survival [23]. IL-7 signaling protects human CD4^+^ effector/memory T cells from apoptosis through up-regulation of Bcl-2 family proteins [24]. Given our observed enrichment of central memory (TCM) and tissue-resident memory (TRM) subsets, these findings are consistent with a cytokine-driven model of memory stabilization within the endometrial niche.

The positive correlations between TRM and both TCM and TEM frequencies observed in our study suggest that tissue-resident and circulating memory pools are co-regulated within the endometrial niche. TRM cells, identified independently by CD69-based gating, represent a non-overlapping population but appear biologically linked to the broader memory hierarchy. Their co-variation with circulating memory subsets may reflect shared maintenance signals such as IL-15 and TGF-β [23,25,26]. In reproductive tissues, TRM have been implicated in rapid local immune surveillance and tolerance regulation [11,12]. Southcombe et al. reported reduced CD69 expression and impaired residency markers in women with recurrent pregnancy loss, suggesting disrupted tissue residency as a mechanism of implantation failure. In contrast, our findings indicate preserved or slightly increased TRM frequencies after miscarriage, which may represent a compensatory or rebound response following prior local inflammation or antigen exposure.

Although terminally differentiated TEMRA subsets did not differ significantly between groups, their correlation patterns revealed distinct relationships with both tissue-resident and central memory compartments. Cytotoxic TEMRA cells (CD8^+^ TEMRA) correlated negatively with cytotoxic tissue-resident memory cells (CD8^+^ TRM), whereas helper-type TEMRA cells (CD4^+^ TEMRA) showed a weak positive correlation with CD8^+^ TRM. In addition, the overall proportion of TEMRA cells was inversely associated with the total tissue-resident memory fraction. These patterns suggest a dynamic equilibrium between terminal effector differentiation and tissue residency, in which increased stability of resident memory cells may limit further effector maturation—or alternatively, persistent antigenic stimulation could promote contraction of the terminally differentiated pool. Such reciprocal balance between terminal effector and resident memory subsets has also been reported in chronic antigen exposure and models of “trained” mucosal immunity [27].

Taken together, our data depict a consistent pattern of endometrial immune remodeling following miscarriage—characterized by expansion of central and resident memory subsets and reduction in effector memory cells. The discriminative capacity of the logistic regression model (AUC = 0.75) supports the biological relevance of this pattern, suggesting that the memory differentiation hierarchy itself, rather than individual subset abundance, encodes a lasting immunological trace of reproductive history. This observation is consistent with reports from chronic infection and vaccine models, where higher TCM/TEM ratios predict enhanced recall potential and tissue responsiveness [28,29]. A central-memory–dominant milieu may bias toward enhanced recall potential with reduced immediate effector reactivity, with plausible consequences for trophoblast interactions and vascular remodeling during implantation.

Mechanistically, several non-exclusive explanations may underlie this remodeling. Pregnancy and subsequent miscarriage may leave persistent antigenic traces or fetal microchimeric cells that maintain low-level stimulation of resident and central memory pools [30,31]. Alternatively, cytokine and stromal signals such as TGF-β, IL-7, and IL-15 could preferentially support TCM and TRM survival while limiting effector differentiation [28,32]. Changes in migration and retention dynamics may also contribute: TEM subsets are highly motile, whereas TCM and TRM display stronger local retention, leading to gradual enrichment of these populations in the post-miscarriage endometrium. Finally, selective attrition of TEMRA cells through apoptosis or exhaustion could stabilize the balance toward memory preservation rather than activation [33,34].

Overall, our findings support the concept that pregnancy loss induces durable restructuring of the local immune memory landscape, influencing subsequent implantation readiness and maternal–fetal tolerance. Strengths of the study include precise timing of biopsies to the implantation window, the use of multicolor flow cytometry on fresh endometrial tissue, and an integrative analytical approach combining correlation, compositional, and regression models. Limitations include reliance on CD69-only gating for TRM (without CD103 confirmation), a cross-sectional design precluding causal inference, and a moderate sample size that limited stratification by miscarriage number. Future studies should incorporate functional assays (cytokine production, proliferation, etc.), TCR sequencing to evaluate clonality, and longitudinal sampling across cycles to assess temporal stability of these immune profiles.

From a clinical perspective, endometrial immune-memory profiling may inform individualized embryo-transfer timing and support the development of targeted immunomodulatory therapies, such as IL-15 or G-CSF-based interventions, aimed at restoring balanced memory-cell homeostasis. If validated, these immune signatures could also serve as diagnostic biomarkers for implantation failure and miscarriage-related endometrial dysfunction. Whether this shift toward central and resident memory enrichment represents adaptive compensation or maladaptive imprinting after miscarriage remains to be determined. Longitudinal follow-up of subsequent implantation outcomes will be required to distinguish between compensatory adaptation and persistent immune dysregulation.

## 5. Conclusions

This study reveals a selective reshaping of the endometrial memory T-cell landscape in women with a history of miscarriage. During the window of implantation, central memory T cells (TCM) were significantly expanded, accompanied by a parallel, though non-significant, increase in tissue-resident memory cells (TRM) and a reduction in effector memory subsets (TEM), while TEMRA frequencies remained stable. These patterns suggest a shift toward a memory profile characterized by long-lived, less differentiated phenotypes, reflecting persistent remodeling of the endometrial immune environment after pregnancy loss. Such central–resident memory enrichment may influence local immune readiness and tolerance during subsequent implantation cycles. Understanding these adaptive changes could be valuable for the development of immune-based diagnostic markers and targeted therapies for miscarriage-related implantation failure.

## Figures and Tables

**Table 1:** Clinical and hormonal characteristics of the study groups (Mean *±* SD).

Variable	Control Group (*n* = 38)	Miscarriage Group (*n* = 33)	*p* Value
Age (years)	41.7 ± 6.8	41.8 ± 5.4	NS
BMI (kg/m^2^)	23.8 ± 2.7	23.7 ± 2.0	NS
Cycle day	19 ± 4	20 ± 6	NS
P4 (ng/mL)	29.7 ± 15.1	27.0 ± 12.2	NS
E2 (pg/mL)	263.8 ± 276.5	184.9 ± 104.2	NS

NS: not significant.

**Table 2:** Distribution of endometrial memory T-cell subsets in women with and without previous miscarriage (Mean ± SD).

Subset	Control (*n* = 38)	Miscarriage (*n* = 33)	*p*-Value
Total memory T cells (% of CD45^+^ lymphocytes)			
TCM	14.5 ± 9.1	20.8 ± 7.7	0.003
TEM	56.8 ± 14.3	48.6 ± 12.9	0.014
TRM	41.7 ± 15.2	47.2 ± 16.7	NS
TEMRA	18.3 ± 8.0	17.5 ± 5.8	NS
CD4^+^ memory T cells (% of CD3^+^CD4^+^)			
TCM	15.0 ± 7.7	13.2 ± 8.8	NS
TEM	70.7 ± 15.5	79.3 ± 11.8	0.011
TRM	30.1 ± 7.7	28.0 ± 7.9	NS
TEMRA	8.1 ± 16.2	4.7 ± 5.7	NS
CD8^+^ memory T cells (% of CD3^+^CD8^+^)			
TCM	20.7 ± 17.1	34.7 ± 19.7	0.002
TEM	50.9 ± 21.7	35.9 ± 18.5	0.003
TRM	39.7 ± 11.2	42.9 ± 11.1	NS
TEMRA	19.5 ± 13.9	18.4 ± 12.5	NS

Notes: Total memory subsets (TCM, TEM, TRM, TEMRA) are expressed as percentages of total CD45^+^ lymphocytes. CD4^+^ and CD8^+^ memory subsets are expressed as percentages within their respective T-cell compartments (CD3^+^CD4^+^ and CD3^+^CD8^+^). NS, not significant.

## Data Availability

The data presented in this study are available on request from the corresponding author due to privacy and ethical requirements.

## Abbreviations

AUC	Area Under the Curve
BMI	Body Mass Index
CD	Cluster of Differentiation
CLR	Centered Log-Ratio
E2	Estradiol
IQR	Interquartile Range
OR	Odds Ratio
P4	Progesterone
PC	Principal Component
PCA	Principal Component Analysis
PERMANOVA	Permutational Multivariate Analysis of Variance
ROC	Receiver Operating Characteristic
SD	Standard Deviation
TRM	Tissue-Resident Memory T cells
TCM	Central Memory T cells
TEM	Effector Memory T cells
TEMRA	Terminally Differentiated Effector Memory T cells
WOI	Window of Implantation
